# Robotic Training and Neural Reorganization in Stroke: A Systematic Review

**DOI:** 10.12688/f1000research.180596.1

**Published:** 2026-05-19

**Authors:** Iyyappan Manickavasagam, Vignesh Srinivasan, Satheeskumar Durairaj, Animesh Hazari, Praveen Kumar Kanadakurti

**Affiliations:** 1 Saveetha College of Physiotherapy, Saveetha Institute of Medical & Technical Science (SIMATS), SIMATS Deemed University, Chennai, Tamil Nadu, India; 2 Dubai Health, Dubai Physiotherapy and Rehabilitation Center, Dubai, United Arab Emirates; 3 College of Health Sciences, Gulf Medical University, Ajman, Ajman, United Arab Emirates

**Keywords:** Exoskeleton Device; Recovery of Function; Locomotion; Motor Skills; Neuroplasticity.

## Abstract

**Introduction:**

Stroke is a primary contributor to global adult morbidity, frequently resulting in debilitating gait disturbances that impede independence. While standard neurorehabilitation remains essential, the advent of robot-assisted gait training (RAGT) provides a platform for high-intensity, reproducible interventions designed to stimulate neuroplasticity. This systematic review evaluated the literature from 2015 to 2025 to determine the influence of RAGT on neural reorganization and functional outcomes in stroke survivors.

**Methodology:**

A systematic search of MEDLINE, PubMed, Scopus, and Cochrane Library was performed for studies published between 2015 and 2025. The inclusion criteria were randomized controlled trials, systematic reviews, and meta-analyses investigating RAGT in acute, subacute, and chronic stroke patients. The evaluation metrics included clinical functional scales (e.g., the Berg Balance Scale and the 10-Meter Walk Test) and neurophysiological markers (e.g., fMRI, fNIRS, QEEG, and BDNF).

**Results:**

Analysis of 23 randomized trials and multiple meta-analyses indicated that RAGT combined with conventional therapy yielded significant improvements in gait speed (standardized mean difference [SMD] = 0.47) and balance (mean difference [MD] = 4.58). Neuroimaging revealed increased activation of the primary motor cortex and supplementary motor areas. Electrophysiological data confirmed reductions in the Power Ratio Index, suggesting normalized cortical activity. End-effector systems have demonstrated superior efficacy in the subacute phase compared to exoskeletons.

**Conclusion:**

RAGT serves as a potent driver of cortical and spinal neuroplasticity by facilitating high-repetition task-specific stimuli. Early intervention during the subacute phase maximizes functional recovery. Future research should prioritize standardizing the dosing and longitudinal monitoring of neural connectivity.

**Trial Registration Status:** Not Applicable.

## Introduction

The global burden of stroke represents one of the most significant challenges to modern healthcare systems, with cerebrovascular accidents serving as the second leading cause of mortality and the third leading cause of long-term motor disability worldwide. ^1^ Among the various sequelae of stroke, gait dysfunction is particularly debilitating, as it fundamentally impairs independence in activities of daily living, restricts social participation, and substantially lowers the perceived quality of life. ^2^ Post-stroke patients often manifest abnormal gait patterns characterized by temporal and spatial asymmetry between steps, reduced propulsion at push-off, and significant postural instability. These deficits are not merely mechanical but reflect a profound disruption of the neural control systems governing locomotion, necessitating a rehabilitation approach that targets the central nervous system’s capacity for reorganization. ^3, 4^

Neuroplasticity, defined as the brain’s ability to modify its structural and functional organization in response to environmental demands and injury, is the biological foundation of stroke recovery. ^5, 6^ This process encompasses a spectrum of changes, ranging from cellular adaptations—such as synaptic strengthening through long-term potentiation and dendritic sprouting—to macroscopic shifts in cortical representation and the re-establishment of interhemispheric balance. ^4, 6^ In the aftermath of a stroke, the brain enters a state of heightened sensitivity to sensorimotor input, particularly during the acute and subacute phases. ^7, 8, 9^ During this “neuroplastic window,” the implementation of intensive, task-specific, and repetitive training is critical for guiding neural reorganization toward functional recovery rather than maladaptive compensation.

Traditional therapist-assisted gait training, while foundational, is often limited by the physical demands placed on clinicians and the inability to provide the high volume of repetitions necessary to trigger robust plastic changes. ^5^ Evidence from animal models suggests that significant synaptic changes in the motor cortex occur only after hundreds of reach movements, and gait training may require 1,000 to 2,000 steps per session to be truly effective. ^5^ In contrast, conventional human physical therapy sessions often achieve fewer than 360 steps, highlighting a substantial “dosage gap” in current clinical practice. ^5^ Robotic-assisted gait training (RAGT) has emerged as a scalable and technology-enhanced solution designed to address these limitations by enabling the delivery of high-intensity, reproducible, and programmable movement patterns which supports Universal Health Coverage. ^2^

The technological evolution of RAGT systems between 2015 and 2025 has been marked by a transition from passive guidance to “assist-as-needed” paradigms and the integration of multimodal feedback systems. ^9^ These systems are broadly categorized into exoskeleton devices, which move joints along a preprogrammed kinematic trajectory, and end-effector systems, which interact with the patient at the distal end of the limb to simulate natural gait cycles. ^7^ Beyond physical assistance, contemporary RAGT often incorporates virtual reality (VR), gaming interfaces, and real-time sensorimotor analytics, all of which enhance patient engagement and cognitive involvement—elements essential for activating the motor learning circuits of the brain. ^9, 10^

While the clinical efficacy of RAGT in improving walking speed and balance has been increasingly validated, the underlying neural mechanisms remain a subject of intense investigation. ^4^ Recent advancements in MRI-compatible robotic devices and portable brain-imaging technologies, such as functional Near-Infrared Spectroscopy (fNIRS) and quantitative Electroencephalography (QEEG), have allowed researchers to observe the cortical and spinal changes induced by robotic therapy in real-time. ^11^ These studies suggest that RAGT facilitates reorganization in both the lesioned and non-lesioned hemispheres, modulates spinal reflex pathways like the H-reflex, and may even influence biochemical markers of plasticity such as Brain-Derived Neurotrophic Factor (BDNF). ^12, 13, 14^

This systematic review aims to provide a comprehensive synthesis of the literature published from 2015 to 2025 regarding the influence of RAGT on neuroplasticity in stroke patients. By analyzing systematic reviews, meta-analyses, and randomized trials, this report evaluates the functional impact of RAGT, identifies the specific neural pathways modulated by robotic interventions, and explores clinical variables, such as timing, device type, and training intensity, that optimize the neuroplastic response. The findings provide evidence-based guidance for integrating robotic technology into standard stroke rehabilitation protocols and highlight future directions for precision neurorehabilitation.

## Methodology

### Study design, literature search and study selection

This systematic review was designed to capture the most significant clinical and technological developments in robotic gait rehabilitation over the past decade. A comprehensive search was conducted across several electronic databases, including MEDLINE, PubMed, the Cochrane Library, Scopus, Web of Science, and Embase. ^2, 9^ The search strategy focused on publications released between January 2015, and December 2025, a period characterized by the maturation of robotic systems and the proliferation of neuroimaging-based trials. ^13^

### Stroke chronicity definitions

In alignment with the Stroke Roundtable Consortium guidelines, recovery phases were categorized as: acute (<7 days), subacute (7 days to 6 months), and chronic (>6 months). These temporal divisions are crucial for interpreting neuroplasticity results, as the brain’s biological receptivity to intervention changes significantly over time. ^15^

### Inclusion and exclusion criteria

Studies were eligible for inclusion if they met the following criteria: (1) adult participants (aged ≥18 years) with a confirmed diagnosis of ischemic or hemorrhagic stroke; (2) interventions involving electromechanical or robotic devices for lower-limb gait or balance training; (3) a comparison group receiving conventional overground gait training or standard-of-care physiotherapy; and (4) the reporting of outcomes related to motor function (e.g., gait speed, balance) or neuroplasticity (e.g., brain activation, connectivity, spinal reflexes). ^2^

Exclusion criteria were applied to studies that focused solely on upper-limb robotics, those lacking a non-robotic control group (unless they were focused on comparing different robotic modalities), and those involving participants with non-vascular brain injuries unless the data for stroke subjects could be clearly disaggregated. ^13^

### Data extraction

Data were extracted from the included studies regarding participant characteristics (age, stroke phase), intervention parameters (type of robot, duration, frequency, intensity), and primary outcomes. ^2^ For the synthesis of neuroplasticity findings, specific attention was paid to the imaging or electrophysiological modalities used and the direction of neural changes reported (e.g., increased activation, enhanced connectivity, normalized frequency spectra). ^6, 16^ The keywords were used with Boolean operators to generate the following MeSH terms: Robotics OR Orthotic Devices AND Recovery of Function AND Locomotion AND Motor Skills AND Neuroplasticity. The eligible articles were titled and abstract screening done by three independent reviewers. The final consensus on the selected articles was taken from an additional two reviewers after full text articles were retrieved. To reduce the risk of bias the study quality was independently assessed on Likert scale ranging from 1 to 10. An average score of 6 was taken as cut-off for final selection of the studies from 5 independent reviewers.

### Ethical considerations

All primary studies included in this review were verified for ethical compliance, including approval from respective Institutional Review Boards and the procurement of informed consent from all human subjects. ^13^ This systematic review, as a synthesis of existing literature, did not require separate ethical approval but adhered to the principles of scientific integrity and transparency in data reporting.

## 3. Results

### 3.1 Functional and clinical outcomes: a meta-analytic synthesis

The collective evidence from 2015 to 2025 demonstrates that RAGT is a powerful adjunct to conventional stroke rehabilitation. The details of study extracts, their inclusion and exclusion are given in the PRISMA flowchart ( Figure 1).

**Figure 1.**
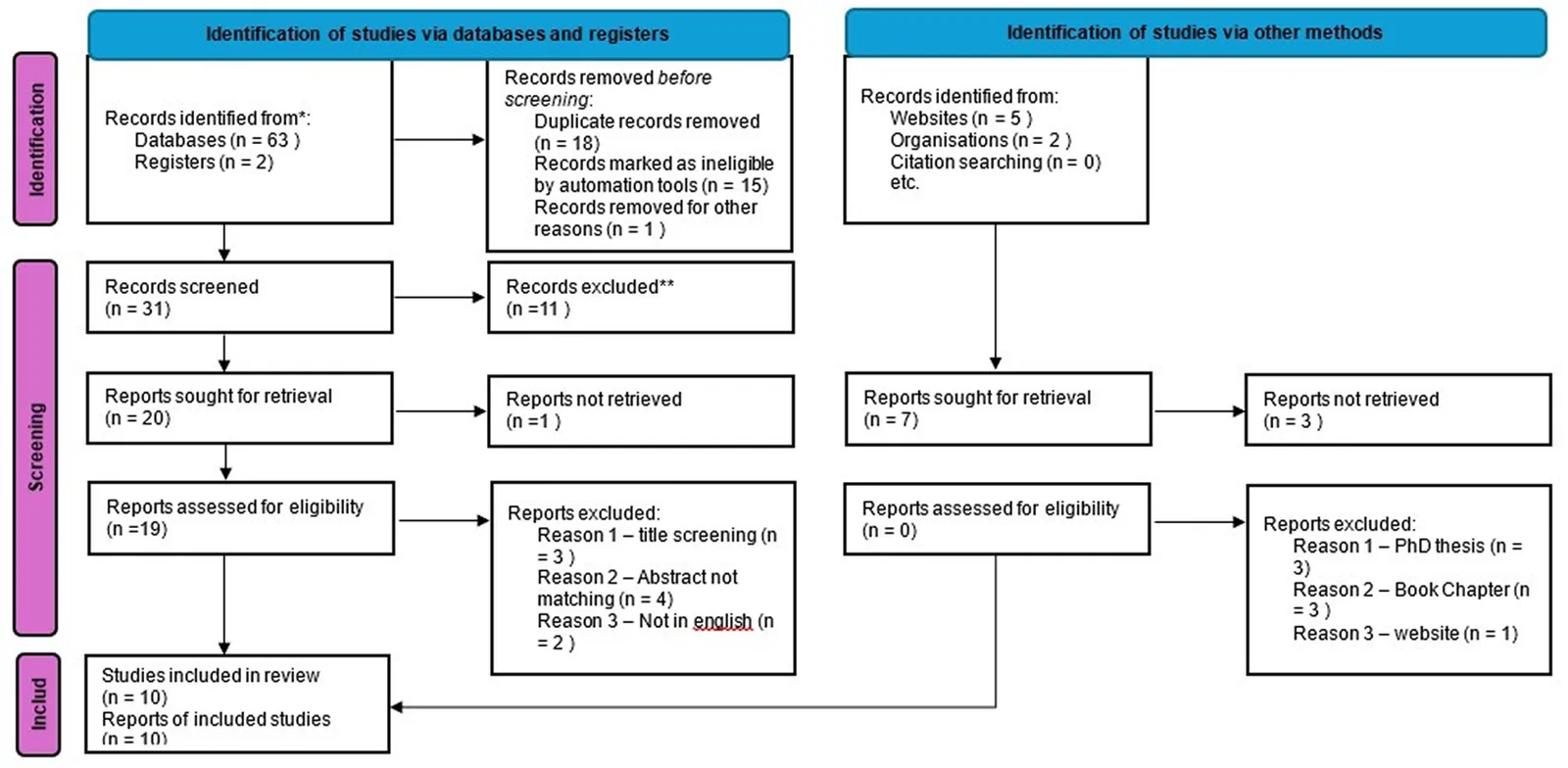
Prisma flow diagram for data extraction.

A 2025 meta-analysis of 23 randomized controlled trials (n = 907) provided the most robust evidence to date regarding functional recovery ^2^ is given in below Table 1.

**Table 1.** Pooled effect sizes of RAGT combined with conventional rehabilitation compared to conventional therapy alone. ^2^

Clinical Outcome	Effect Size (SMD/MD)	P-Value	Heterogeneity (I ^2^)
Gait Function (e.g., FAC)	SMD = 0.51	0.001	73%
Gait Speed (10MWT)	SMD = 0.47	0.010	62%
Balance (Berg Balance Scale)	MD = 4.58 points	< 0.001	2%
Activities of Daily Living (FIM/ADL)	SMD = 0.35	0.001	39%

FAC: Functional Ambulation Categories; 10MWT: 10-Meter Walk Test; SMD: Standardized Mean Difference; I ^2^: Heterogeneity;

The exceptionally low heterogeneity for balance improvements (I ^2^ = 2%) indicates that robotic systems provide a highly consistent and reliable stimulus for postural stability across diverse patient populations. ^2^ The improvements in gait speed were most pronounced when interventions were limited to ≤15 sessions, suggesting that early gains in kinematics may plateau without subsequent increases in training complexity or duration. ^2^

### 3.2 Neuroimaging and cortical activation markers

A central focus of research during the 2015–2025 period was the identification of neuroimaging biomarkers that correlated with RAGT-induced recovery. Studies using fNIRS and fMRI have consistently identified specific regions of the motor cortex that respond to robotic stimuli. ^6^

3.2.1 Regional activation patterns

Robotic training facilitates a more robust activation of the motor-related cortices in the affected hemisphere compared to conventional overground walking. This reorganization is particularly evident in the primary motor cortex (M1), the supplementary motor area (SMA), and the premotor cortex (PMC) ^6^ which is described in below Table 2.

**Table 2.** Key cortical regions modulated by RAGT as identified by fNIRS and fMRI. ^6^

Brain Region	Mechanism of Change	Clinical Correlation
Ipsilesional M1	Increased metabolic activation (beta-values)	Improved motor execution and limb control
Supplementary Motor Area (SMA)	Enhanced neural activity and planning capacity	Improved ability to plan and sequence movement6
Premotor Cortex (PMC)	Increased connectivity and cortical thickness	Enhanced complex movement modulation6
Prefrontal Cortex (Bilateral)	Increased metabolic demand during early RAGT	Heightened cognitive engagement and attention6

fMRI: functional Magnetic Resonance Imaging; fNIRS: functional Near-Infrared Spectroscopy; M1: Primary Motor Cortex; SMA: Supplementary Motor Area; PMC Premotor Cortex;

3.2.2 Functional network topology

Recent studies leveraging graph theory analysis of fMRI data have shown that RAGT promotes “whole-brain” network reorganization. ^2, 6^ Successful robotic interventions are associated with the normalization of network metrics, such as a shortened average path length and improved global efficiency. ^6, 16^ These changes suggest that the brain’s functional network reverts toward “small-world” properties, which are characterized by high local aggregation and efficient long-distance communication. ^16^

### 3.3 Electrophysiological findings: EEG and QEEG

Quantitative EEG (QEEG) has emerged as a valuable tool for measuring the “cortical preparatory state” and spectral power changes after robotic therapy. A significant finding across multiple studies is the reduction of pathological slow-wave activity. ^6^

Power Ratio Index (PRI) and Delta/Alpha Ratio (DAR): RAGT significantly reduces the PRI—calculated as (delta + theta)/(alpha + beta)—and the DAR in the PMC, SMA, and M1. These reductions are negatively correlated with Fugl-Meyer Assessment (FMA) scores, indicating that as the electrophysiological frequency spectrum moves toward high-frequency (alpha/beta) dominance, motor function improves. ^6^

Alpha-Band Coherence: A 2025 study demonstrated that RAGT, especially when combined with electroacupuncture, enhances coherence between the somatosensory association cortex (electrode CZ) and motor planning areas (FCZ, FC2). ^12^ This enhanced connectivity suggests a more efficient transfer of sensory information to motor planning centers.

Event-Related Desynchronization (ERD): Changes in ipsilesional beta-band ERD and synchronization (ERS) have been observed following RAGT, reflecting the normalization of cortical excitability during voluntary leg movements. ^17^

### 3.4 Spinal plasticity and reflex modulation

The neuroplastic effects of RAGT extend to the spinal cord, where repetitive stepping patterns influence the locomotor circuitry.

H-Reflex Modulation: Body weight-supported RAGT has been shown to re-establish the physiological phase modulation of the soleus H-reflex. ^18^ In healthy individuals, the H-reflex is depressed during the swing phase to allow for ankle dorsiflexion; in stroke patients, this modulation is often lost, leading to spasticity and foot drop. RAGT helps restore this depression at the stance-to-swing transition, fostering a more natural step progression. ^18^

Operant Conditioning: Studies on volitional down-regulation of the H-reflex show that stroke survivors can achieve an 80% success rate in reflex suppression when given real-time robotic feedback. ^18^ This down-regulation is strongly correlated with improvements in gait symmetry and velocity. ^18^

### 3.5 Biochemical markers: brain-derived neurotrophic factor (BDNF)

BDNF is a primary mediator of activity-dependent neuroplasticity, yet its response to RAGT in human stroke patients remains complex.

Circulating Levels: Systematic reviews suggest that aerobic exercise and functional tasks with RAGT promote changes in central BDNF concentrations, although serum levels may not always reflect these shifts. ^19^ A subtle but consistent negative influence is observed in patients carrying the Val66Met polymorphism, who may exhibit slower rates of learning and functional recovery. ^1^

Peripheral Epigenetic Changes: Recent research has identified that while circulating protein levels may remain stable, resisted training with RAGT and aerobic exercise can induce differential DNA methylation of gene in paretic skeletal muscle, potentially regulating the local expression of growth factors. ^20^

### 3.6 Comparative efficacy: end-effector vs. exoskeleton

The architectural design of the robotic system significantly influences the neuroplastic and functional outcomes, as given in the below Table 3.

**Table 3.** Comparison of functional and mechanistic advantages between end-effector and exoskeleton systems. ^2^

Device Type	Mechanism	Superior Outcomes
End-Effector (e.g., Loko help, Gait Trainer)	Distal drive (feet); free hip/knee movement	Higher rates of independent walking; superior in subacute phase; consistent gait speed gains. ^2^
Exoskeleton (e.g., Lokomat)	Proximal drive (joints); rigid kinematic guidance	Better for non-ambulatory/severe patients; improved proximal muscle control; high variability in gait speed. ^2^

RAGT: Robot-Assisted Gait Training

End-effector systems allow for greater movement variability and “destabilization training,” which forces the patient to engage in active motor planning and error correction—two critical components of neuroplasticity. ^21^ Conversely, exoskeletons are superior for early mobilization of severely impaired patients who require total assistance to maintain an upright posture.

## 4. Discussion

### 4.1 The repetition threshold and the “dosage” of plasticity

The findings from 2015 to 2025 reinforce the fundamental principle of neurorehabilitation: recovery is a dose-dependent process. The primary advantage of robotic systems is their ability to bridge the “repetition gap” that exists in conventional therapy. ^5^ Data from end-effector-based RAGT shows that patients increase their step count from an average of 1,098 in the first session to over 1,500 in the final session—a 39% increase in training volume over a standard six-week program. ^5^ This high volume of stepping is essential for overcoming the threshold required to induce long-term potentiation in the motor cortex. ^5^

Furthermore, the “assist-as-needed” paradigm—where the robot only provides support when the patient’s performance falls below a certain threshold—has been shown to be more effective than continuous passive guidance. ^21^ This is because neuroplasticity is driven by the correlation between volitional motor intent and sensory feedback. ^16^ Passive movement alone may maintain joint range of motion, but it fails to trigger the significant metabolic and electrical changes in the motor planning areas (SMA and PMC) seen during active robotic training. ^6^

### 4.2 Top-down vs. bottom-up neuroplasticity

The effectiveness of RAGT can be understood through two complementary mechanisms. The “bottom-up” approach utilizes repetitive, high-volume sensory input from the lower limbs to reorganize spinal circuits and stimulate the somatosensory cortex. ^16, 21^ By simulating a near-normal gait cycle with standardized plantar pressure and proprioceptive feedback, RAGT reinforces the existing neural pathways and promotes the recruitment of alternative pathways (e.g., the corticospinal tract from the non-lesioned hemisphere). ^16^

The “top-down” approach targets the brain’s executive and planning functions. By integrating RAGT with virtual reality (VR), clinicians can manipulate the visual and cognitive environment to demand higher levels of attention and spatial awareness. ^9, 10^ Studies have shown that VR-enhanced RAGT leads to greater improvements in walking speed and balance-related gait metrics compared to robotic training alone, likely due to the activation of prefrontal and parietal regions involved in multisensory integration. ^9^

### 4.3 The subacute phase: the critical window for intervention

The timing of rehabilitation initiation is the most critical clinical modifier of neuroplasticity. The strongest effect sizes for RAGT (SMD = 0.74 to 0.75) are found in the acute and subacute phases, while chronic stroke survivors show more limited, though still significant, improvement (SMD = 0.23). This disparity highlights the “neuroplastic window”—a period of several months post-stroke where the brain is biochemically and structurally more receptive to reorganization. ^9^

Early RAGT initiation (within 3 months of onset) has been shown to accelerate bi-hemispheric reorganization in motor-related brain regions and reduce the length of stay in rehabilitation facilities. ^12^ In contrast, in the chronic stage, neuroplasticity is often exhausted or stabilized, requiring more intensive or longer-duration interventions (e.g., >6 weeks) to achieve meaningful functional changes. ^8^

### 4.4 Combined interventions and synergistic plasticity

One of the most promising trends in the last decade is the combination of RAGT with other neuromodulatory techniques such as tDCS.

Non-invasive Brain Stimulation (NIBS): Combining RAGT with repetitive Transcranial Magnetic Stimulation (rTMS) or Transcranial Direct Current Stimulation (tDCS) has shown synergistic effects. Specifically, low-frequency rTMS applied to the unaffected hemisphere can reduce maladaptive interhemispheric inhibition, “priming” the affected hemisphere to respond more effectively to the sensorimotor training provided by the robot. ^4^

Electroacupuncture (EA): The integration of EA with RAGT has been shown to improve the functional connectivity of the brain network more effectively than either treatment alone. The findings suggest that EA may reverse the pathological frequency spectrum imbalance seen after stroke, aiding the neural network in re-entering an efficient information-processing state. ^12^

### 4.5 Clinical limitations and practical implications

Despite the evidence for its neuroplastic potential, several barriers to the widespread adoption of RAGT remain.

1. Heterogeneity of Protocols: There is currently no clear consensus on the optimal “dose” of RAGT. Trial protocols vary widely in terms of session duration (30 to 60 minutes), frequency (3 to 5 times per week), and total sessions (15 to 40). ^22^
2. Cost and Accessibility: The high up-front and ongoing costs of robotic systems must be balanced against long-term gains in patient independence and the reduction of caregiver burden. ^10^ Reducing the high upfront costs is essential for ensuring that these innovative health infrastructures are available to diverse patient populations, thereby reducing the long-term societal cost of disability.
3. Chronic Phase Efficacy: While RAGT can benefit chronic patients, the magnitude of the effect is smaller, suggesting that “maintenance” or “booster” protocols may be needed to sustain initial gains. ^3^
4. Individualized Response: Genetic factors, such as the BDNF Val66Met polymorphism, and clinical variables like the location and extent of the brain lesion, contribute to high inter-patient variability in the neuroplastic response. ^1^

## Conclusion

The literature from 2015 to 2025 established robot-assisted gait training as a cornerstone of technology-enhanced neurorehabilitation. RAGT facilitates significant functional recovery in stroke survivors, particularly in the domains of balance and gait function, by providing a high-volume task-specific stimulus that far exceeds the intensity of conventional physical therapy. This mechanical stimulus is translated into neurological reorganization through the activation of key motor planning areas, normalization of cortical frequency spectra, and modulation of spinal locomotor circuits.

The findings underscore the importance of early intervention, as the subacute phase represents the most fertile ground for neuroplastic changes. Furthermore, the evidence supports a nuanced approach to device selection, with end-effector systems showing particular effectiveness in improving gait speed and independence, whereas exoskeletons remain vital for the mobilization of severely impaired individuals. As the field progresses, the integration of RAGT with adjunct technologies, such as neuromodulation and virtual reality, alongside the use of advanced biomarkers to tailor interventions, will likely be the next step in achieving personalized and profound neurological restitution for stroke survivors. The integration of RAGT represents a significant step toward personalized and sustainable neurorehabilitation. By optimizing the timing and dosage of intervention, clinicians can contribute to the global goal of enhancing well-being and functional independence for the growing population of stroke survivors. Future research must focus on determining the optimal longitudinal dosage and long-term sustainability of these robotically induced neural changes.

## Ethics approval and consent to participate

The study was conducted in line with the Declaration of Helsinki and approved by the Dubai Scientific Research Ethics Committee (DSREC), Dubai Health Authority (MBRU IRB-2024-417), Dubai, UAE, for ethical clearance.

## Consent to participate

Not Applicable.

## Data Availability

Figshare. RTNS-REVIEW. https://doi.org/10.6084/m9.figshare.32064228. ^23^ The project contains the following underlying data:
• DSREC-DECISION_Student_MBRU IRB-2024-417 [Ethical Approval Letter] •
PRISMA_2020_checklist Data is available under the terms of the CC BY 4.0.

## References

[1] De Assis GG Murawska-Ciałowicz E : Brain-derived neurotrophic factor and stroke: Perspectives on exercise as a health care strategy. *Human Movement.* 2024;25(1):1–14. 10.5114/hm.2024.136050

[2] Lee JH Kim G : Effectiveness of robot-assisted gait training in stroke rehabilitation: A systematic review and meta-analysis. *J. Clin. Med.* 2025;14(13):4809. 40649182 10.3390/jcm14134809 PMC12250684

[3] Bonanno M De Pasquale P Lombardo Facciale A : May patients with chronic stroke benefit from robotic gait training with an end-effector? A case-control study. *Journal of Functional Morphology and Kinesiology.* 2025;10(2):161. 40407445 10.3390/jfmk10020161 PMC12101270

[4] Jehangir A Ahmad I Feng Y : Combine effects of robotic assisted gait training with transcranial magnetic stimulation on gait metrics and balance in stroke patients: A pilot randomized control. *Front. Neurol.* 2025;16:1655409. 41356236 10.3389/fneur.2025.1655409 PMC12675174

[5] Pichardo A Malovec M : Enhancing post-stroke gait rehabilitation with robot-assisted therapy: A focus on step repetitions and neuroplasticity. *Archives of Clinical and Biomedical Research.* 2025;9(3). 10.26502/acbr.50170453

[6] Li X Zhang H Zhang W : Neural mechanisms underlying the improvement of gait disturbances in stroke patients through robot-assisted gait training based on QEEG and fNIRS: A randomized controlled study. *J. Neuroeng. Rehabil.* 2025;22. 40533805 10.1186/s12984-025-01656-2 PMC12175367

[7] Kim H Park G Shin J-H : Neuroplastic effects of end-effector robotic gait training for hemiparetic stroke: A randomised controlled trial. *Sci. Rep.* 2020;10(1):Article 12461. 10.1038/s41598-020-69367-3 PMC7385173 32719420

[8] Morone G Paolucci S Cherubini A : Robot-assisted gait training for stroke patients: Current state of the art and perspectives of robotics. *Neuropsychiatr. Dis. Treat.* 2017;13:1303–1311. 28553117 10.2147/NDT.S114102 PMC5440028

[9] Amirbekova M Kispayeva T Adomaviciene A : Systematic review and meta-analysis of effectiveness of robotic therapy in the recovery of motor functions after stroke. *Front. Hum. Neurosci.* 2025;19. 40761519 10.3389/fnhum.2025.1622661 PMC12318999

[10] Yang J Gong Y Yu L : Effect of exoskeleton robot-assisted training on gait function in chronic stroke survivors: A systematic review of randomised controlled trials. *BMJ Open.* 2023;13(9):Article e074481. 10.1136/bmjopen-2023-074481 PMC10503387 37709309

[11] Berger A Horst F Müller S : Current state and future prospects of EEG and fNIRS in Robot-assisted gait rehabilitation: A brief review. Front. Hum. Neurosci. 2019;13 10.1136/bmjopen-2023-074481 PMC6561323 31231200

[12] Huang H Su X Zhang Y : Effect of robot-assisted gait training combined with electroacupuncture on lower limb motor function and brain network characteristics in stroke: An EEG study. *J. Neuroeng. Rehabil.* 2025;22. 41430331 10.1186/s12984-025-01827-1 PMC12751702

[13] Kim DH Kang CS Kyeong S : Robot-assisted gait training promotes brain reorganization after stroke: A randomized controlled pilot study. *NeuroRehabilitation.* 2020;46(4):483–489. 32508339 10.3233/NRE-203054

[14] Stevenson AJ Mrachacz-Kersting N Asseldonk E van : Spinal plasticity in robot-mediated therapy for the lower limbs. *J. Neuroeng. Rehabil.* 2015;12:81. 26377324 10.1186/s12984-015-0073-x PMC4574007

[15] Bernhardt J Hayward KS Kwakkel G : Agreed definitions and a shared vision for new standards in stroke recovery research: The Stroke Recovery and Rehabilitation Roundtable taskforce. *Neurorehabil. Neural Repair.* 2017;31(9):793–799. 10.1177/1545968317732668 28934920

[16] Magouni M Astrakas LG Elbach S : Integrated neuroimaging and robotic rehabilitation in chronic stroke: Neural correlates and predictors of motor recovery. *Exp. Ther. Med.* 2025;30(4):1–14. 40787596 10.3892/etm.2025.12932 PMC12329399

[17] Heng HM Lu MK Chou LW : Changes in balance, gait and electroencephalography oscillations after robot-assisted gait training: An exploratory study in people with chronic stroke. *Brain Sci.* 2022;12(8):Article 1054. 10.3390/brainsci10110821 PMC7694605 33171938

[18] Tahayori B Koceja D : Exercise induced operant conditioning of the H-reflex in stroke patients: Hopes for improving motor function through inducing plastic changes in the spinal pathways. *Journal of Neurology, Neurological Science and Disorders.* 2019;5(1):001–004. 10.17352/jnnsd.0000026

[19] Alcantara CC García-Salazar LF Silva-Couto MA : Post-stroke BDNF concentration changes following physical exercise: A systematic review. *Front. Neurol.* 2018;9. 30210424 10.3389/fneur.2018.00637 PMC6121011

[20] Plaza-Diaz J Izquierdo D Torres-Martos Á : Impact of physical activity and exercise on the epigenome in skeletal muscle and effects on systemic metabolism. *Biomedicine.* 2022;10(1):126. 35052805 10.3390/biomedicines10010126 PMC8773693

[21] Lou Z Wang F Guan D : Effect of end-effector robot training on lower limb motor function and balance in stroke patients: A systematic review and meta-analysis. *Front. Neurol.* 2026;16:Article 1752104. 10.3389/fneur.2025.1752104 PMC12827104 41586090

[22] Sarı A : Comparison of end-effector and exoskeleton devices with robot-assisted gait training in patients with stroke. *Journal of Surgery and Medicine.* 2021;5(2):128–131. 10.28982/josam.877434

[23] Durairaj SK : RTNS-REVIEW.Dataset. *figshare.* 2026. 10.6084/m9.figshare.32064228.v2

