# Supplementary material for: Robotic Training and Neural Reorganization in Stroke: A Systematic Review
Source: F1000Res. 2026 May 19;15:762. [Version 1] doi: 10.12688/f1000research.180596.1 (PMC13620352; doi:10.12688/f1000research.180596.1)
Supplement: Peer review history

# Reviewer report: Reviewer response for version 1

**DOI:** 10.5256/f1000research.199215.r522014

**Published:** 2026-09-28

**Authors:** Rajan Balakrishnan

**Affiliations:** 1 MAHSA University Malaysia, Selangor, Malaysia

Description and Relevance:

 The manuscript titled **"Robotic Training and Neural Reorganization in Stroke: A Systematic Review"** (Manickavasagam et al., 2026) explores the impact of Robot-Assisted Gait Training (RAGT on neuroplasticity and functional recovery in adult stroke survivors. Synthesizing literature from 2015 to 2025, the review pair’s clinical functional outcomes such as gait speed, balance and activities of daily living with underlying neurophysiological, electrophysiological, spinal and biochemical mechanisms including fMRI, fNIRS, QEEG, H-reflex, and BDNF.

 This review is highly relevant to neurorehabilitation clinicians, researchers and biomedical engineers. It bridges the gap between technological interventions and central nervous system remodeling. It provides actionable insights into factors like training timing in subacute and chronic phases.

 Overview of key findings

1. **Clinical & Functional Improvements:** Meta-analytic data cited in the review shows that combining RAGT with conventional therapy significantly improves gait speed, balance, Functional Ambulation Categories and ADL outcomes. The balance outcomes demonstrated remarkably low heterogeneity.
2. **Cortical & Network Reorganization:** Neuroimaging (fNIRS/fMRI) highlights increased activation in the primary motor cortex (ipsilesional M1), supplementary motor area (SMA), premotor cortex (PMC) and bilateral prefrontal cortex. Graph theory analysis demonstrates a shift toward small-world network properties with enhanced global efficiency.
3. **Electrophysiological & Spinal Markers:** QEEG findings demonstrate a reduction in pathological slow-wave indices in Power Ratio Index and Delta/Alpha Ratio on motor areas. At the spinal level, RAGT helps re-establish physiological soleus H-reflex phase modulation during the stance-to-swing transition.
4. **Subacute Window & Device Efficacy:** The strongest neuroplastic effect sizes occur during the acute and subacute phases. End-effector devices show superior gains in independent walking speed and active motor planning due to greater movement variability whereas exoskeletons are better suited for severely impaired, non-ambulatory individuals needing rigid guidance. 

**Major Points:**

**Inconsistency in Methodological Reporting vs. PRISMA Diagram:**

  In Section 2 ( *Data extraction*), the text states that study quality was assessed on a Likert scale (1–10) with an average score of 6 used as a cutoff. However, standard systematic review frameworks typically use validated tools like the PEDro scale, Cochrane Risk of Bias tool, or QUADAS. Furthermore, the PRISMA flow diagram (Figure 1) presents discrepancies that need clarification:

1.
  - Identification: Databases n=63, Registers n=2.
  - Screening: Reports assessed for eligibility n=19; 9 excluded (leaving 10 included).
  - Results section: Section 3.1 references a 2025 meta-analysis of 23 randomized controlled trials (n=907) as part of its pooled effect size table (Table 1). Clarify whether this manuscript is a systematic review of primary literature (n=10 included studies) or umbrella review/meta-synthesis incorporating secondary meta-analyses.

**Dose-Response and Longitudinal Sustainability:**

 The finding that gains in gait speed plateaued when interventions were limited to 15 sessions is crucial. However, the manuscript would benefit from a deeper critique of how long these neuroplastic and functional changes persist post-intervention. Adding a dedicated subsection or table discussing follow-up retention rates (e.g., 3-month or 6-month follow-ups) across the included studies would significantly strengthen the clinical recommendations. 

**Mechanistic Depth on BDNF & Epigenetics:**

1. Section 3.5 outlines peripheral BDNF levels and epigenetic changes (DNA methylation in paretic muscle). Given the conflicting evidence regarding circulating serum BDNF vs. central concentrations, the authors should explicitly synthesize how genotype variations (such as the Val66Met polymorphism) alter responsiveness to RAGT, providing a clearer framework for personalized neurorehabilitation. 

**Minor points:**

**PRISMA Figure Readability & Formatting:**

 Image resolution for Figure 1 is low, making text within boxes difficult to read clearly. Re-rendering Figure 1 as a vector graphic or high-resolution image will improve readability.

 In Figure 1 under excluded reports, "Not in english" contains a minor typographical error.

**Table Footnotes and Abbreviations:**

 In Table 1, ensure all abbreviations in the legend e.g., FAC, 10MWT, SMD match all metrics presented in the table columns.

 In Table 3, the "Mechanism" cell for *Exoskeleton* appears with a formatting extra line break before "Proximal drive (joints)". 

**Synthesis of Combined Modalities:**

 Section 4.4 discusses synergistic plasticity when combining RAGT with Transcranial Direct Current Stimulation (tDCS), repetitive Transcranial Magnetic Stimulation (rTMS) or Electroacupuncture. A brief mention of optimal sequencing (e.g., priming with NIBS prior to RAGT vs. concurrent application) would add practical value for clinicians.

Are the rationale for, and objectives of, the Systematic Review clearly stated?

Yes

Is the statistical analysis and its interpretation appropriate?

Yes

If this is a Living Systematic Review, is the ‘living’ method appropriate and is the search schedule clearly defined and justified? (‘Living Systematic Review’ or a variation of this term should be included in the title.)

Yes

Are sufficient details of the methods and analysis provided to allow replication by others?

Yes

Are the conclusions drawn adequately supported by the results presented in the review?

Yes

Reviewer Expertise:

Physiotherapy

I confirm that I have read this submission and believe that I have an appropriate level of expertise to confirm that it is of an acceptable scientific standard.

## References

[1] : Robot-assisted gait training in stroke. *Turkish Journal of Physical Medicine and Rehabilitation* .2024;70(3) : 10.5606/tftrd.2024.15681293-299 10.5606/tftrd.2024.15681 PMC1163949639679124

# Reviewer report: Reviewer response for version 1

**DOI:** 10.5256/f1000research.199215.r493939

**Published:** 2026-07-08

**Authors:** S. Jeyakumar Jeyakumar

**Affiliations:** 1 Garden City University, Bangalore, Karnataka, India

Recommendation: Kindly accept this manuscript for indexing.

1. The manuscript addresses a highly relevant and timely topic by comprehensively reviewing the role of robot-assisted gait training in promoting neuroplasticity and functional recovery after stroke, making it valuable for clinicians, rehabilitation specialists, and researchers.
2. The review is well organized and methodologically sound. The objectives, inclusion criteria, literature search strategy, and presentation of findings are clearly described, providing a transparent and systematic synthesis of the available evidence.
3. The discussion successfully integrates clinical outcomes with neurophysiological mechanisms. The inclusion of functional, neuroimaging, electrophysiological, spinal, and biochemical evidence offers a comprehensive understanding of how RAGT contributes to post-stroke recovery.
4. The manuscript demonstrates strong clinical relevance. The comparison of different robotic systems, emphasis on intervention timing, and discussion of future directions provide practical guidance that can support evidence-based neurorehabilitation.
5. The manuscript is well written, scientifically balanced, and supported by recent literature. The conclusions are consistent with the presented evidence and highlight important implications for future research and clinical practice.

Overall Recommendation: The manuscript is of good scientific quality, provides a comprehensive and up-to-date review of the literature, and makes a meaningful contribution to the field of stroke rehabilitation. I recommend acceptance for indexation.

Are the rationale for, and objectives of, the Systematic Review clearly stated?

Yes

Is the statistical analysis and its interpretation appropriate?

Yes

If this is a Living Systematic Review, is the ‘living’ method appropriate and is the search schedule clearly defined and justified? (‘Living Systematic Review’ or a variation of this term should be included in the title.)

Yes

Are sufficient details of the methods and analysis provided to allow replication by others?

Yes

Are the conclusions drawn adequately supported by the results presented in the review?

Yes

Reviewer Expertise:

Geriatrice rehabilitationa and stroke rehabilitation.

I confirm that I have read this submission and believe that I have an appropriate level of expertise to confirm that it is of an acceptable scientific standard.
